# A key regulatory loop AK4P1/miR-375/SP1 in pancreatic adenocarcinoma

**DOI:** 10.1080/15592294.2022.2148433

**Published:** 2022-12-07

**Authors:** Wangjin Xu, Weiyang Lou, Linhang Mei

**Affiliations:** a Department of Oncological Surgery, Taizhou Hospital of Zhejiang Province Affiliated to Wenzhou Medical University, Taizhou, 317000, China; b Department of Breast Surgery, The First Affiliated Hospital, College of Medicine, Zhejiang University, Hangzhou, 310003, China

**Keywords:** AK4P1, miR-375, SP1, pancreatic adenocarcinoma, prognosis

## Abstract

Pancreatic adenocarcinoma is one of the leading lethal human cancer types and is notorious for its poor prognosis. A series of bioinformatic analyses and experimental validations were employed to explore the role and mechanism of pseudogene-derived RNAs in pancreatic adenocarcinoma. Consequently, a total of 13 upregulated and 7 downregulated pseudogene-derived RNAs in pancreatic adenocarcinoma were identified. Survival analysis revealed a statistically predictive role of AK4P1 for unfavourable prognosis of patients with pancreatic adenocarcinoma. Subcellular location analysis indicated that AK4P1 was mainly located in cytoplasm, in which AK4P1 might competitively bind to tumour suppressive miR-375 in pancreatic adenocarcinoma. Further analysis showed that SP1 was a potential downstream target gene of miR-375 in pancreatic adenocarcinoma. Intriguingly, expression determination validated that SP1 could positively regulate AK4P1 levels in pancreatic adenocarcinoma. Finally, AK4P1 might also exert its effects by interacting with oncogenic parental gene AK4 in pancreatic adenocarcinoma. Conclusively, the present study elucidated a key regulatory loop AK4P1/miR-375/SP1 in pancreatic adenocarcinoma.

## Introduction

Pancreatic adenocarcinoma, the most common type of pancreatic malignancy, ranks the seventh leading cause of cancer-related deaths [1]. During the past decades, a large number of advances in the aspects of diagnosis, pre-operation management, surgical treatment, and systematic therapy for pancreatic adenocarcinoma have been achieved [2], the patients with pancreatic adenocarcinoma still possess poor prognosis and rapidly aggress, with nearly 100% of five-year mortality rate [3]. Therefore, it is an urgent need to explore the molecular mechanism and seek effective therapeutic targets or promising prognostic biomarkers in pancreatic adenocarcinoma.

Pseudogene-derived RNAs are a group of non-coding Rs, which play key role in occurrence and progression of human disorders, including cancer [4]. Among all action mechanisms of pseudogene-derived RNAs, competing endogenous RNA (ceRNA) hypothesis has been widely acknowledged and reported [5]. For example, Hou *et al*. suggested that KRT17P3 promoted cisplatin resistance by modulating miR-497-5p/mTOR in non-small cell lung cancer [6]; Zheng *et al*. indicated that the 3’UTR of CYP4Z2P enhanced tumour angiogenesis of breast cancer by acting as a ceRNA for CYP4Z1 [7]; Zheng *et al*. demonstrated that pseudogene-derived RNAs were involved in regulation of GJA1 by competitively binding to miR-30d-5p in pancreatic cancer [8]. To date, a systematic study regarding the expression, prognosis, function, and molecular mechanism of pseudogene-derived RNAs in pancreatic adenocarcinoma is still absent and needs to be further elucidated.

In this study, we firstly performed differential expression analysis for pseudogene-derived RNAs in pancreatic adenocarcinoma, then conducted survival analysis for those significant differentially-expressed pseudogene-derived RNAs in pancreatic adenocarcinoma and next focused on the molecular mechanism of a pseudogene-derived RNA named AK4P1 in pancreatic adenocarcinoma. Finally, a key regulatory loop AK4P1/miR-375/SP1 was identified in pancreatic adenocarcinoma.

## Results

### Identification of AK4P1 as a potential pseudogene-derived RNA in PAAD

**Figure 1.**
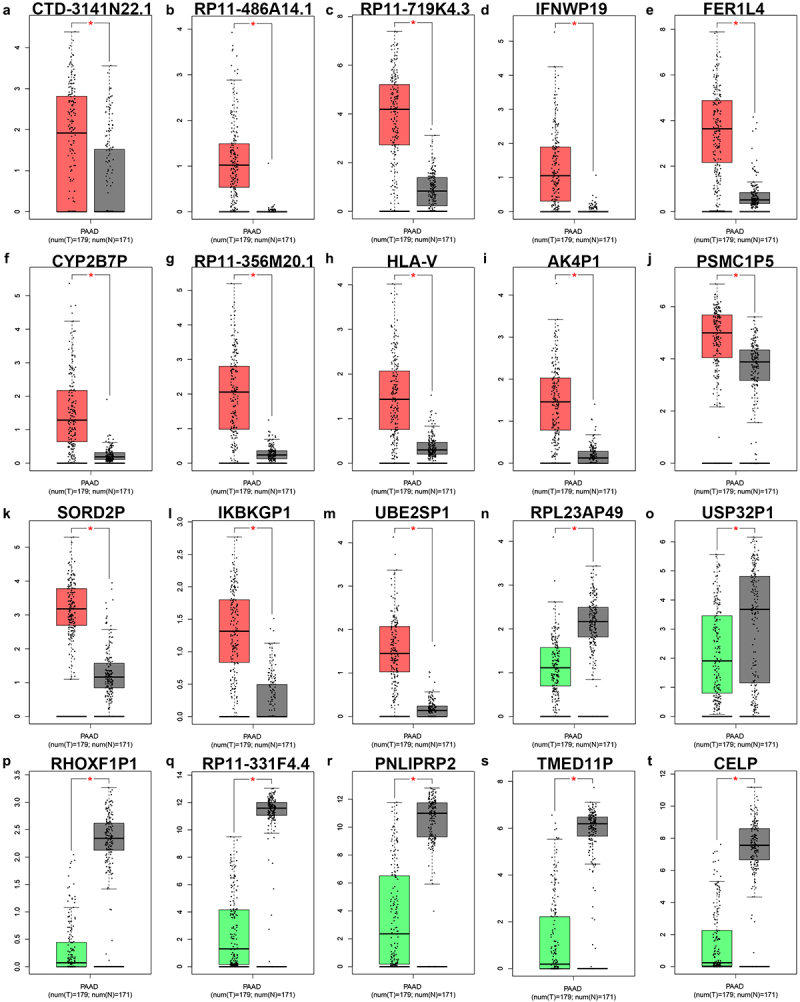
The expression of significant differentially expressed pseudogene-derived RNAs in PAAD. The expression levels of CTD-3141N22.1 (a), RP11-486A14.1 (b), RP11-719K4.3 (c), IFNWP19 (d), FER1L4 (e), CYP2B7P (f), RP11-356M20.1 (g), HLA-V (h), AK4P1 (i), PSMC1P5 (j), SORD2P (k), IKBKGP1 (l), UBE2SP1 (m), RPL23AP49 (n), USP32P1 (o), RHOXF1P1 (p), RP11-331F4.4 (q), PNLIPRP2 (r), TMED11P (s) and CELP (t) in PAAD determined by GEPIA database. *P-value<0.05.

**Figure 2.**
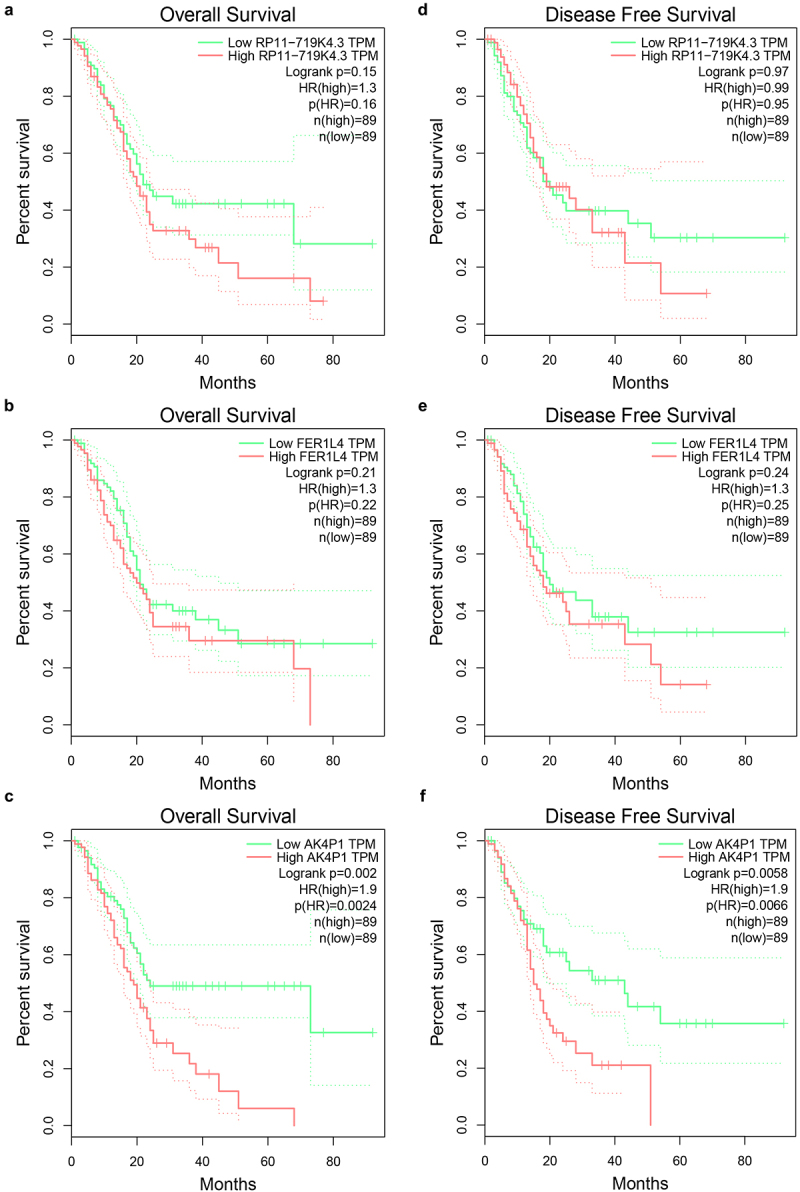
Survival analysis for potential pseudogene-derived RNAs in PAAD determined by GEPIA database. The prognostic values (overall survival) of RP11-719K4.3 (a), FER1L4 (b) and AK4P1 (c) in PAAD. The prognostic values (disease free survival) of RP11-719K4.3 (A), FER1L4 (B) and AK4P1 (C) in PAAD.

In order to identify potential pseudogene-derived RNAs in PAAD, dreamBase database was employed. Finally, a total of 621 significant differentially expressed pseudogene-derived RNAs in PAAD were found out, consisting of 258 upregulated and 363 downregulated RNAs (**Table S1**). To improve the analytic accuracy, GEPIA database was introduced to verify the expression levels of the 621 pseudogene-derived RNAs in PAAD. Among these RNAs, 13 upregulated and 7 downregulated pseudogene-derived RNAs were identified as shown in Figure 1. After performing stage expression analysis, 3 of 20 pseudogene-derived RNAs presented significant expression differences among various major stages in PAAD, involving RP11-719K4.3, FER1L4, and AK4P1 (Table 1 and **Figure S1**). Subsequently, survival analysis was conducted to assess the prognostic values of the three RNAs in PAAD. Two prognostic indices, consisting of overall survival and disease-free survival, were included. As suggested in Figure 2, among the three RNAs, only high expression of AK4P1 indicated poor overall survival and disease-free survival in PAAD. Taken together, AK4P1 might be a potential oncogenic RNA molecule and a promising prognostic biomarker in PAAD.

**Table 1.** The expression differences of pseudogenes among various major stages in PAAD analysed by GEPIA database.

Name	F-value	P-value
CTD-3141N22.1	0.063	0.979
RP11-486A14.1	0.501	0.682
RP11-719K4.3	4.620	0.004
IFNWP19	0.631	0.596
FER1L4	3.530	0.016
CYP2B7P	1.480	0.221
RP11-356M20.1	1.240	0.298
HLA-V	0.905	0.440
AK4P1	4.810	0.003
PSMC1P5	2.210	0.089
SORD2P	1.880	0.135
IKBKGP1	1.190	0.313
UBE2SP1	0.848	0.470
RPL23AP49	0.330	0.804
USP32P1	0.138	0.937
RHOXF1P1	2.170	0.094
RP11-331F4.4	1.650	0.180
PNLIPRP2	0.968	0.409
TMED11P	1.430	0.235
CELP	1.570	0.199

### miR-375 is a potential binding miRNA of AK4P1 in PAAD

**Figure 3.**
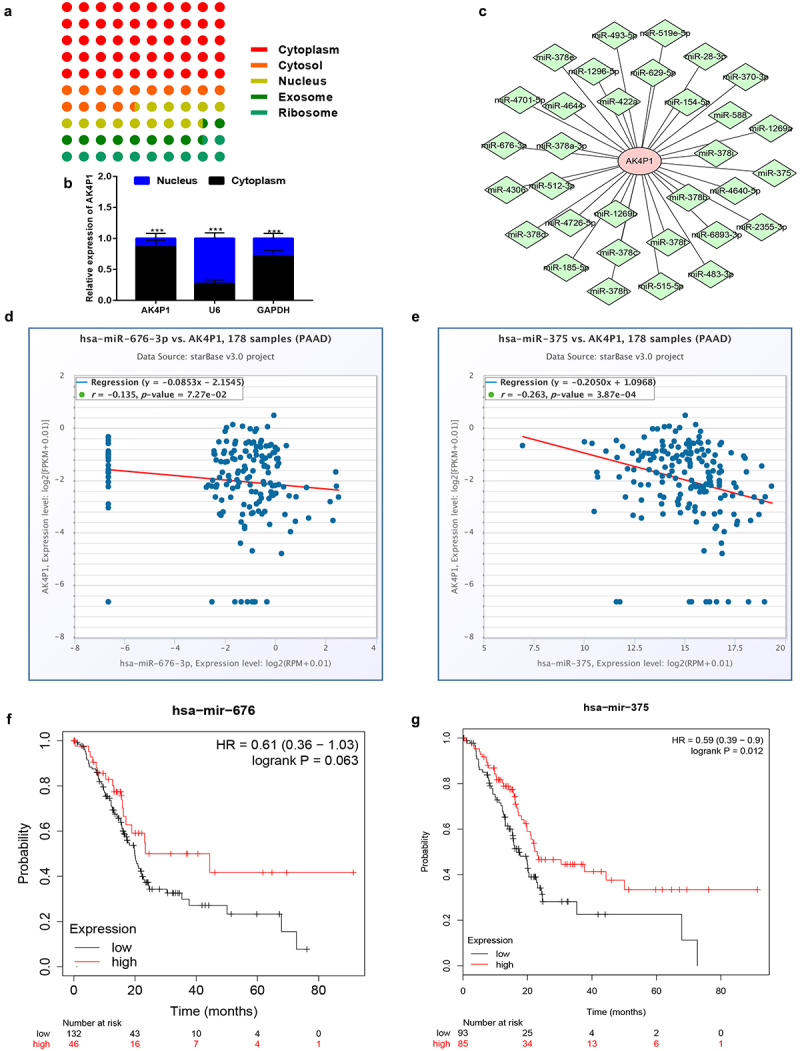
Identification of miR-375 as a downstream binding miRNA of AK4P1 in PAAD. The subcellular location of AK4P1 analysed by lncLocator database (a) and nucleo-cytoplasm separation assay (b). (c) The AK4P1-miRNA regulatory network established by Cytoscape software. The expression correlation of AK4P1 with miR-676-3p (d) or miR-375 (e) in PAAD determined by starBase database. The prognostic values of miR-676-3p (f) and miR-375 (g) in PAAD analysed by Kaplan-Meier plotter database.

As previously reported, cytoplasm-located pseudogene-derived RNAs can regulate gene expression by competitively binding to shared miRNAs [5,8–10]. Therefore, we analysed the subcellular location of AK4P1 using lncLocator database. As shown in Figure 3a, AK4P1 was mainly located in cytoplasm, which was followingly validated by nucleo-cytoplasm separation assay (Figure 3b). Subsequently, the downstream miRNAs of AK4P1 were predicted by starBase database. A total of 32 binding miRNAs were found out and a regulatory AK4P1-miRNA network was established using Cytoscape software (Figure 3c). The expression correlation of AK4P1 with the 32 miRNAs was determined (Table 2). As presented in Figure 3 d and e, only two miRNAs (miR-676-3p and miR-375) were negatively correlated with AK4P1 expression in PAAD. Besides, the prognostic values of miR-676-3p and miR-375 were evaluated by Kaplan–Meier plotter database (figure 3 f and g). The result indicated that PAAD patients with high expression of miR-375 had favourable prognosis, but no significant prognostic predictive role for miR-676-3p in PAAD was observed. These findings suggest that miR-375 might be the most potential downstream binding miRNA of AK4P1 in PAAD.

**Table 2.** The correlation analysis between AK4P1 and predicted miRNA in PAAD analysed by starBase database.

miRNA	pseudogene	R-value	P-value
miR-4644	AK4P1	0.032	0.673
miR-185-5p	AK4P1	−0.004	0.962
miR-4306	AK4P1	0.020	0.793
miR-515-5p	AK4P1	−0.033	0.659
miR-519e-5p	AK4P1	−0.064	0.393
miR-4701-5p	AK4P1	0.013	0.859
miR-588	AK4P1	0.001	0.989
miR-512-3p	AK4P1	0.088	0.242
miR-2355-3p	AK4P1	0.319	0.000
miR-676-3p	AK4P1	−0.135	0.073
miR-28-3p	AK4P1	0.322	0.000
miR-154-5p	AK4P1	0.017	0.823
miR-493-5p	AK4P1	0.026	0.728
miR-378c	AK4P1	0.263	0.000
miR-378 f	AK4P1	−0.049	0.518
miR-378a-3p	AK4P1	0.173	0.021
miR-422a	AK4P1	0.000	1.000
miR-378 h	AK4P1	0.000	1.000
miR-378i	AK4P1	0.021	0.783
miR-378b	AK4P1	0.139	0.064
miR-378d	AK4P1	0.187	0.013
miR-378e	AK4P1	0.000	1.000
miR-370-3p	AK4P1	0.030	0.692
miR-6893-3p	AK4P1	0.000	1.000
miR-629-5p	AK4P1	0.164	0.029
miR-483-3p	AK4P1	0.044	0.560
miR-375	AK4P1	−0.263	0.000
miR-4640-5p	AK4P1	0.118	0.115
miR-4726-5p	AK4P1	0.036	0.630
miR-1296-5p	AK4P1	−0.030	0.694
miR-1269a	AK4P1	−0.016	0.831
miR-1269b	AK4P1	0.039	0.602

### SP1 is a downstream target gene of miR-375 in PAAD

**Figure 4.**
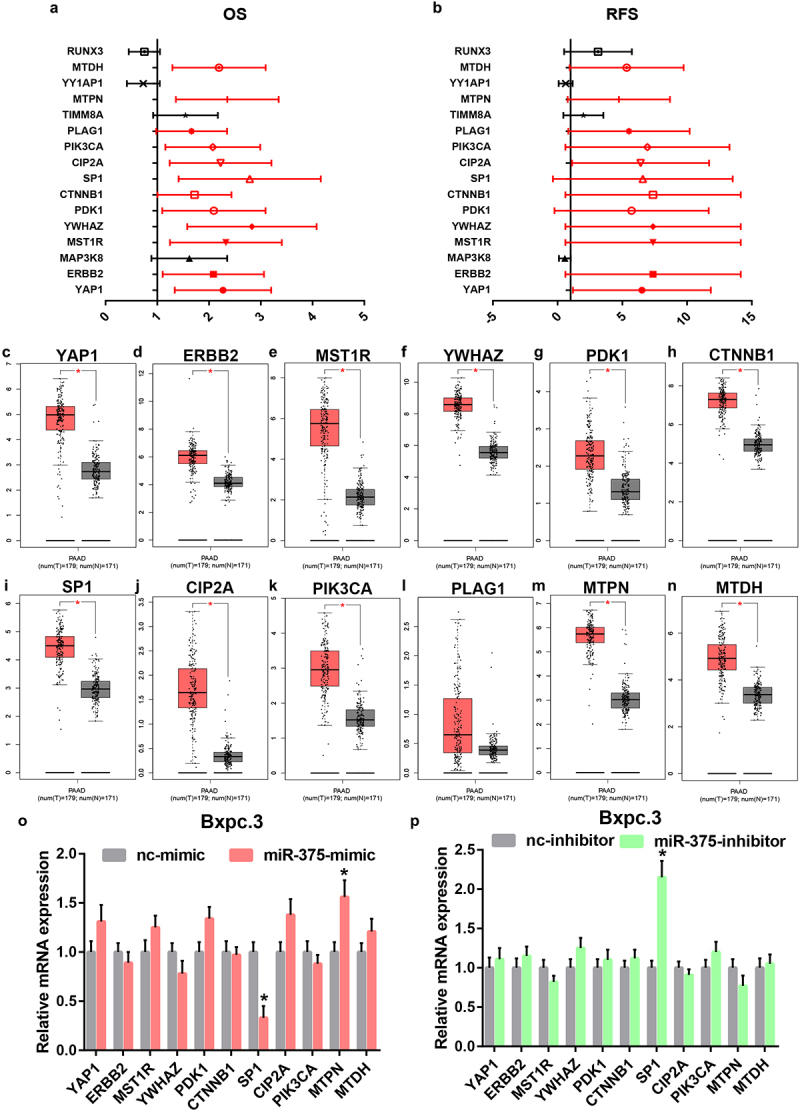
Identification of SP1 as a potential downstream target gene of miR-375 in PAAD. (a) The overall survival analysis for the target genes of miR-375 in PAAD. (b) The relapse free survival analysis for the target genes of miR-375 in PAAD. The mRNA expression levels of YAP1 (c), ERBB2 (d), MST1R (e), YWHAZ (f), PDK1 (g), CTNNB1 (h), SP1 (i), CIP2A (j), PIK3CA (k), PLAG1 (l), MTPN (m) and MTDH (n) in PAAD when compared with normal controls. (o) The mRNA expression changes of 12 target genes after miR-375 overexpression in PAAD cell line Bxpc.3. (p) The mRNA expression changes of 12 target genes after miR-375 inhibition in PAAD cell line Bxpc.3. *P-value<0.05.

As widely acknowledged, miRNAs exert their biological roles by negatively modulating gene expression. Therefore, the potential target genes of miR-375 were predicted by miRNet database. To screen out the most potential target genes, only 31 miR-375-target gene pairs that have been validated by reporter assay were included in this study. Based on the action mechanism of miRNA, there should be a negative expression correlation of miR-375 with target genes in PAAD. Thus, correlation analysis was performed by usage of TCGA PAAD data. As listed in Table 3, 16 target genes were significantly inversely correlated with miR-375 in PAAD. Next, survival analysis for the 16 target genes in PAAD was conducted. As suggested in Figure 4 a and b, among the 16 target genes, only increased expression of 12 genes (YAP1, ERBB2, MST1R, YWHAZ, PDK1, CTNNB1, SP1, CIP2A, PIK3CA, PLAG1, MTPN, and MTDH) was positively linked to poor OS and RFS in PAAD. Subsequently, the expression levels of the 12 target genes in PAAD were determined by GEPIA database (Figure 4c–n). Intriguingly, 11 target genes were markedly upregulated in PAAD when compared with normal tissues but no statistical difference of PLAG1 was observed. Correlation analysis revealed that the 11 genes were statistically positively associated with AK4P1 expression in PAAD (**Figure S2**). Next, we intended to ascertain if miR-375 could regulate these target genes in PAAD. After transfection of miR-375 mimic, only SP1 expression was markedly decreased in PAAD cell line Bxpc.3 (Figure 4o). And by administration of miR-375 inhibitor, among these target genes, only SP1 expression was significantly upregulated in Bxpc.3 (Figure 4p). Taken these findings into consideration, miR-375 could negatively regulate SP1 expression in PAAD.

**Table 3.** The correlation analysis of miR-375 and predicted target genes in PAAD determined by starBase database.

ID	Target	R-value	P-value
miR-375	YAP1	−0.481	0.000
miR-375	ERBB2	−0.424	0.000
miR-375	MAP3K8	−0.388	0.000
miR-375	MST1R	−0.367	0.000
miR-375	YWHAZ	−0.345	0.000
miR-375	PDK1	−0.342	0.000
miR-375	CTNNB1	−0.297	0.000
miR-375	SP1	−0.292	0.000
miR-375	CIP2A	−0.289	0.000
miR-375	PIK3CA	−0.256	0.001
miR-375	PLAG1	−0.254	0.001
miR-375	TIMM8A	−0.241	0.001
miR-375	MTPN	−0.231	0.002
miR-375	YY1AP1	−0.209	0.005
miR-375	MTDH	−0.189	0.012
miR-375	RUNX3	−0.151	0.044
miR-375	TP53	−0.147	0.050
miR-375	IGF1R	−0.106	0.157
miR-375	MALAT1	−0.080	0.291
miR-375	LRP5	−0.065	0.386
miR-375	USP1	−0.062	0.409
miR-375	LDHB	−0.030	0.690
miR-375	FZD8	0.044	0.556
miR-375	JAK2	0.048	0.524
miR-375	ADIPOR2	0.057	0.452
miR-375	C1QBP	0.101	0.178
miR-375	MYCN	0.135	0.073
miR-375	DEPTOR	0.155	0.039
miR-375	PHLPP1	0.238	0.001
miR-375	RASD1	0.551	0.000
miR-375	ELAVL4	0.731	0.000

### Transcription factor SP1 is responsible for AK4P1 overexpression in PAAD

**Figure 5.**
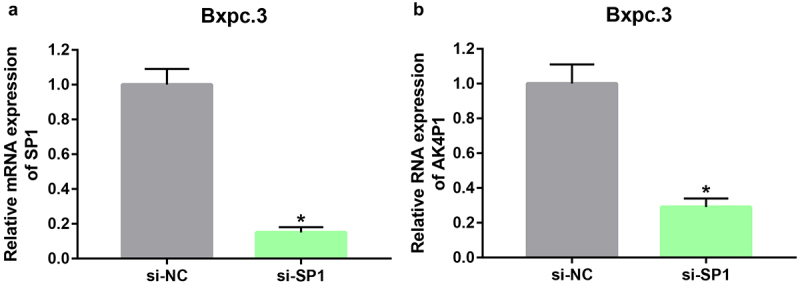
SP1 enhances AK4P1 expression in PAAD. (a) SP1 is significantly positively correlated with AK4P1 in PAAD. (b) SP1 is overexpressed in PAAD when compared with normal tissues. *P-value<0.05.

Using online database prediction, 40 transcription factors of AK4P1 were forecasted. Subsequently, expression correlation analysis of AK4P1 with predicted transcription factors was performed (**Table S2**). Among these transcription factors, SP1 was significantly positively linked to AK4P1 expression in PAAD, with the correlation coefficient equal to 0.51. Next, we inhibited SP1 expression by transfection of the specific siRNA targeting SP1 in PAAD cell line Bxpc.3 (Figure 5a). As suggested in Figure 5b, AK4P1 expression was markedly decreased after knockdown of SP1 in PAAD. Intriguingly, knockdown of AK4P1 could also significantly downregulate SP1 protein expression level in PAAD cell (**Figure S3**). These results showed that transcription factor SP1 activation might be a key mechanism responsible for AK4P1 upregulation in PAAD.

### AK4 is a potential parental gene of AK4P1 in PAAD

**Figure 6.**
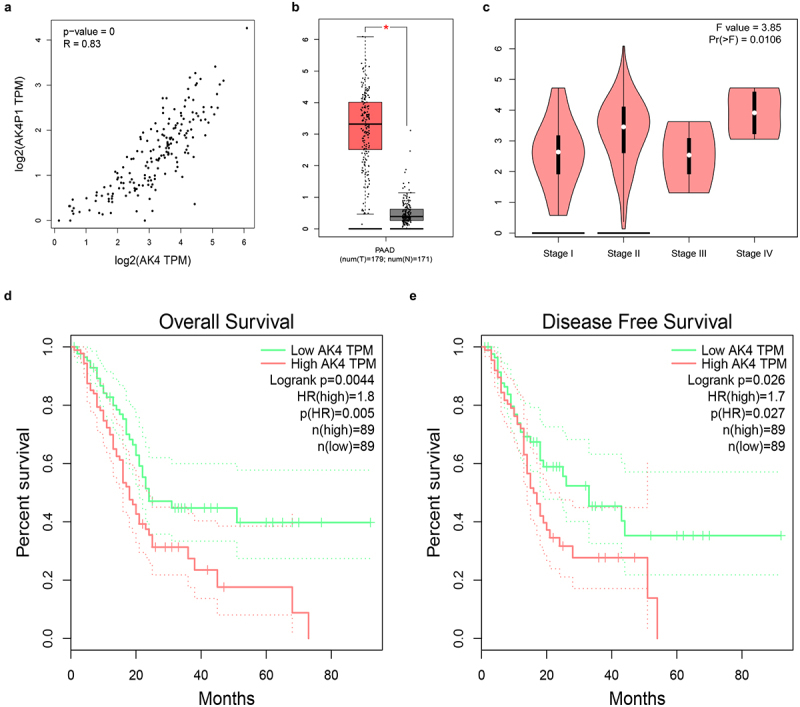
AK4 is a potential parent gene of AK4P1 in PAAD. (a) AK4P1 is significantly positively linked to AK4 expression in PAAD. (b) AK4 expression is markedly upregulated in PAAD when compared with normal tissues. (c) The expression differences of AK4 among various major stage in PAAD. (d) The prognostic value (overall survival) of AK4 in PAAD. (e) The prognostic value (disease free survival) of AK4 in PAAD. *P-value<0.05.

Pseudogene-derived RNAs can also exert their roles by positively regulating corresponding parental genes. AK4 is the parental gene of AK4P1. Correlation analysis revealed that AK4 was significantly positively associated with AK4P1 in PAAD (Figure 6a). Moreover, expression analysis indicated a marked upregulation of AK4 in PAAD when compared with normal tissues (Figure 6b). Next, stage expression analysis for AK4 in PAAD was performed. The result showed that the statistical expression difference of AK4 among various stages in PAAD was observed (Figure 6c). Furthermore, AK4 presented significant prognostic values (overall survival and disease-free survival) in PAAD as suggested in Figure 6 d and e. Moreover, knockdown of AK4P1 could also significantly downregulate AK4 protein expression level in PAAD cell (Figure S3). Taken together, AK4 is a potential parental gene of AK4P1, and AK4P1 might positively regulate AK4 in PAAD.

## Discussion

Pancreatic adenocarcinoma is notorious for its poor prognosis. It makes sense to elucidate the molecular action mechanism of pathogenesis and progression of pancreatic adenocarcinoma.

In this study, we first performed differential expression analysis for pseudogene-derived RNAs in pancreatic adenocarcinoma using dreamBase database, after which GEPIA database was employed to validated those RNAs’ expression levels. Consequently, 13 upregulated pseudogene-derived RNAs (CTD-3141N22.1, RP11-486A14.1, RP11-719K4.3 *et al*.) and 7 downregulated pseudogene-derived RNAs (RPL23AP49, USP32P1, RHPXF1P1 *et al*.) were identified in pancreatic adenocarcinoma. Some of these pseudogene-derived RNAs have been reported to be involved in the development and progression of human cancers. For instance, FER1L4 played critical roles in multiple malignancies, including papillary thyroid cancer [11], colorectal cancer [12], liver cancer [13], oral squamous cell carcinoma [14]. Next, stage expression analysis for the 20 RNAs was conducted in pancreatic adenocarcinoma by GEPIA database, and three RNAs (RP11-719K4.3, FER1L4 and AK4P1) were selected for subsequent study. Among the three RNAs, only AK4P1 possessed statistical prognostic values (overall survival and disease-free survival) in pancreatic adenocarcinoma. These findings suggest that AK4P1 might be an oncogenic pseudogene-derived RNA and a promising prognostic biomarker in pancreatic adenocarcinoma.

As a type of noncoding RNA, pseudogene-derived RNA could exert its effects through competitively binding to shared miRNAs [15,16]. To ascertain if AK4P1 functions by this way in pancreatic adenocarcinoma, first of all, lncLocator tool was used to predict the subcellular location of AK4P1 as previously described [17]. The result, together with the finding from nucleo-cytoplasm separation assay, showed that AK4P1 mainly located in cytoplasm, which provided the suitable place for AK4P1-miRNA binding. Using starBase analysis, 32 miRNAs (miR-28-3p, miR-154-5p, miR-588 *et al*.) were predicted to potentially bind to AK4P1. Among these miRNAs, only two miRNAs, involving miR-676-3p and miR-375, were inversely correlated with AK4P1 expression in pancreatic adenocarcinoma. Survival analysis showed that miR-375 was a significant favourable prognostic biomarker in pancreatic adenocarcinoma. Lots of studies have demonstrated that miR-375 was a tumour suppressive miRNA in human cancers. For example, miR-375 inhibited progression of hepatocellular carcinoma by targeting JAK2/STAT3 signalling pathway [18]; miR-375 reduced the stemness of gastric cancer cells through triggering ferroptosis [19]; miR-375 also functioned as a tumour suppressor in tongue squamous cell carcinoma [20]. These reports together with our previous analytic results suggested that miR-375 might be a downstream binding miRNA of AK4P1 in pancreatic carcinoma.

Subsequently, we explored the downstream action mechanism of AK4P1/miR-375 in pancreatic adenocarcinoma. It has been widely acknowledged that miRNAs play crucial biological functions by suppressing target gene expression and function [21,22]. By combination of target gene prediction, correlation analysis, survival analysis, and expression determination, a total of 11 potential target genes, involving YAP1, ERBB2, MST1R, YWHAZ, PDK1, CTNNB1, SP1, CIP2A, PIK3CA, PLAG1, MTPN, and MTDH, were screened in pancreatic adenocarcinoma. Among these target genes, only SP1 expression could be negatively regulated by miR-375 in pancreatic adenocarcinoma. SP1 is a well-known transcription factor which is implicated in an ample variety of essential biological processes and have been confirmed critical for cell growth, apoptosis, death, and carcinogenesis [23]. Previous studies have indicated that transcription factors could activate expression of ncRNA, including pseudogene-derived RNA. For example, Zheng *et al*. suggested that transcription factor E2F1-induced FTH1P3 accelerated gefitinib resistance of non-small cell lung cancer [24]; Yang *et al*. demonstrated that transcription factor STAT1-induced upregulation of lncRNA LINC01123 predicted poor prognosis and enhanced the progression of endometrial cancer by miR-516b/KIF4A [25]. Intriguingly, AK4P1 expression was significantly decreased after knockdown of SP1 in pancreatic adenocarcinoma. Our findings suggested that AK4P1 increased SP1 expression by competitively binding to shared miR-375 and SP1 could conversely positively regulate AK4P1 expression in pancreatic adenocarcinoma.

Our team and other groups have validated that pseudogene-derived RNAs could exert their biological effects by alteration of expression and function of parental genes [9,26]. Thus, we explored the relationship between AK4P1 and its parental gene AK4 in pancreatic adenocarcinoma. AK4 has been reported to act as an oncogene in several types of human cancer, including ovarian cancer [27], lung cancer [28,29], HER2-positive breast cancer [30] and bladder cancer [31]. A positive correlation of AK4P1 with AK4 in pancreatic adenocarcinoma was observed. Expression determination, stage expression detection, and survival analysis revealed that AK4 was significantly upregulated in pancreatic carcinoma tissues and its high expression predicted favourable prognosis in pancreatic carcinoma. Moreover, knockdown of AK4P1 could significantly decrease AK4 expression in PAAD. These findings indicated that AK4P1 might positively regulate its parental gene AK4, thereby exerting its roles in pancreatic carcinoma.

**Figure 7.**
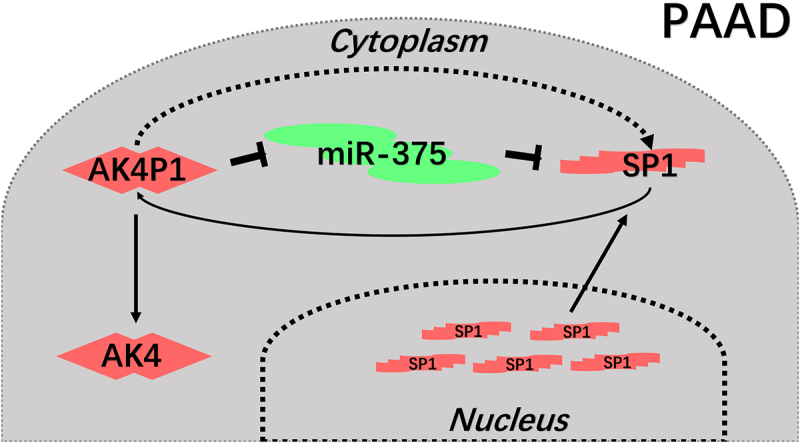
The action model of AK4P1 in PAAD.

In conclusion, the present study elucidated a key regulatory loop AK4P1/miR-375/SP1 in pancreatic carcinoma (Figure 7). Every member in this axis possessed significant values for predicting prognosis of patients with pancreatic adenocarcinoma. Moreover, we also demonstrated that AK4P1 might also exert its oncogenic effects through positively regulating AK4 in pancreatic carcinoma. However, these findings need to be further confirmed by much more experimental validation and clinical trials in the future.

## Materials and methods

### dreamBase analysis

RNA-seq expression data of pseudogene-derived RNAs in human PAAD and normal pancreatic tissues (from TCGA database) were downloaded from the dreamBase database (http://rna.sysu.edu.cn/dreamBase/index.php) which is a comprehensive platform for analysing modulatory characteristics of pseudogene-derived RNAs from multi-dimensional high-throughput sequencing data [32]. |log_2_(Fold change) (Tumour)|>1.0 was set as the cut-off criterion for identifying differentially expressed pseudogene-derived RNA in PAAD. These selected pseudogene-derived RNAs were considered as candidate RNAs.

### GEPIA analysis

Gene Expression Profiling Interactive Analysis (http://gepia.cancer-pku.cn/detail.php), also named GEPIA, is an integrated interactive database that provides customizable functions, including differential expression, stage expression, survival analysis, and correlation analysis [33]. In this study, GEPIA was employed to confirm expression of candidate pseudogene-derived RNAs (cancer tissues: TCGA PAAD samples; normal tissues: TCGA normal pancreatic tissues and GTEx normal pancreatic tissues), determine expression differences of pseudogene-derived RNAs among various major stage (TCGA PAAD data), assess prognostic values of pseudogene-derived RNAs, analyse target gene expression, evaluate expression correlation for RNA–RNA pairs in PAAD. *P*-value<0.05 was considered as statistically significant.

### lncLocator analysis

lncLocator (http://www.csbio.sjtu.edu.cn/bioinf/lncLocator/index.html), a lncRNA subcellular localization predictor based on a stacked ensemble classifier, was employed to analyse the subcellular location of AK4P1 [34].

### starBase analysis

starBase (http://starbase.sysu.edu.cn/), an open-source database for investigating the miRNA-ncRNA, miRNA-mRNA, ncRNA-RNA, RNA–RNA, RBP-ncRNA, and RBP–mRNA interactions from CLIP-seq, degradome-seq, and RNA–RNA interactome data [35,36] was used to predict binding miRNAs of AK4P1 and analyse RNA–RNA expression correlation in TCGA PAAD.

### miRNet analysis

miRNet (http://www.mirnet.ca) is a miRNA-centric network visual analytics platform, which was introduced to predict target genes of miR-375 [37,38]. Only miR-375-target gene pairs validated by reporter assay were included for subsequent analysis.

### Kaplan-Meier plotter analysis

The prognostic values of miRNAs and target genes in PAAD were assessed using Kaplan–Meier plotter (http://kmplot.com/analysis), which is capable to access the effect of miRNA, gene or protein on survival in more than 20 different cancer types, including PAAD. Logrank *P*-value <0.05 was considered as statistically significant.

### Cell culture

The human PAAD cell line Bxpc.3 was obtained from China Center for Type Culture Collection. Bxpc.3 was cultured in RPMI-1640 (Biological Industries) supplemented with 10% foetal bovine serum (Biological Industries) and placed at 37°C in a humidified incubator containing 5% CO_2_.

### Cell transfection

The specific small interfering RNA (siRNA) targeting SP1 (si-SP1) and negative control (si-NC) were designed and synthesized by Guangzhou Ribobio Co. Ltd. (Guangzhou, China). miR-375 mimic, mimic control, inhibitor, and inhibitor control were also obtained from Guangzhou Ribobio Co. Ltd. (Guangzhou, China). Bxpc.3 cells were seeded into six-well plates, after which transfection was performed using Lipofectamine^TM^ 3000 (Invitrogen, Shanghai, China) according to the manufacturer’s instruction.

### RNA extraction and RT-qPCR

The total RNA was extracted from cells using RNAiso plus Reagent (TaKaRa, Kusatsu, Japan). Then, total RNA was reversely transcribed into complementary DNA (cDNA) by the PrimeScript RT Reagent Kit (TaKaRa, RR0037A). Next, qPCR was conducted using SYBR Premix Ex Taq (TaKaRa, RR420A) in a Roche LightCycler480 II Real-Time PCT Detection System. The gene expression levels relative to GAPDH were analysed using the method of 2^−ddCt^.

### Western blot analysis

The protein levels of AK4 and SP1 in PAAD cells were determined by normalization to GAPDH as we previously described [39].

### Statistical analysis

In this study, most of statistical analyses were performed by corresponding online databases. Besides, experiments were conducted in triplicates and the results were presented as mean ± standard deviation (SD). The experimental statistical analysis was performed using GraphPad Prism Software (Version 7). The differences between two groups were assessed by a two-tailed Student’s *t*-test. *P*-value <0.05 was considered as statistically significant.

## Supplementary Material

Supplemental Material
