# Supplementary material for: A key regulatory loop AK4P1/miR-375/SP1 in pancreatic adenocarcinoma
Source: Epigenetics. 2022 Dec 7;18(1):2148433. doi: 10.1080/15592294.2022.2148433 (PMC9980666; doi:10.1080/15592294.2022.2148433)
Supplement: Supplemental Material [file KEPI_A_2148433_SM4859.docx]

**Supplements**

**Table S1**. The differentially expressed pseudogenes identified by dreamBase database.

| Name | Fold change |
| --- | --- |
| CTD-3141N22.1 | 7.73 |
| RP11-848P1.7 | 6.32 |
| ZBTB45P1 | 6.23 |
| RP11-1223D19.2 | 5.86 |
| IGHV5-78 | 4.95 |
| RP11-402P6.13 | 4.95 |
| RP11-392E22.5 | 4.81 |
| CYMP | 4.81 |
| OR7E91P | 4.64 |
| RP11-309L24.6 | 4.54 |
| AC002075.4 | 4.52 |
| COX20P1 | 4.25 |
| RPS26P47 | 4.17 |
| RP11-64D22.5 | 4.00 |
| RP11-343H5.4 | 4.00 |
| MTND4P20 | 3.98 |
| RP11-333E13.2 | 3.91 |
| AC092569.2 | 3.91 |
| AL590762.11 | 3.91 |
| RPL12P28 | 3.91 |
| GTF2IP9 | 3.81 |
| ECEL1P2 | 3.76 |
| RP11-459D22.1 | 3.70 |
| PTMAP3 | 3.59 |
| KRT16P2 | 3.59 |
| RP11-613E4.5 | 3.59 |
| RP11-757O6.6 | 3.59 |
| RP11-486A14.1 | 3.53 |
| RPL29P33 | 3.46 |
| RPS15AP11 | 3.32 |
| RP11-430K21.2 | 3.32 |
| RP11-766H1.1 | 3.32 |
| RP11-1012A1.7 | 3.32 |
| RP11-480N24.4 | 3.32 |
| IGHV1OR15-3 | 3.32 |
| AMYP1 | 3.32 |
| RPL23AP77 | 3.32 |
| RP11-565J7.1 | 3.32 |
| AC007000.10 | 3.17 |
| AC090954.5 | 3.17 |
| RP11-719K4.3 | 3.14 |
| KRT16P1 | 3.00 |
| RP11-2C24.5 | 3.00 |
| AC096579.13 | 3.00 |
| RP1-179N16.3 | 3.00 |
| AC005740.3 | 3.00 |
| AC110299.5 | 2.81 |
| MTND4P19 | 2.81 |
| RP11-732A19.1 | 2.81 |
| RP11-579D7.8 | 2.81 |
| RPL12P27 | 2.81 |
| AC098828.3 | 2.81 |
| RP11-161H23.10 | 2.81 |
| CPHL1P | 2.68 |
| RPS10P3 | 2.59 |
| RP11-154P18.2 | 2.59 |
| PPIAP19 | 2.59 |
| RP11-173A8.2 | 2.59 |
| CH507-42P11.2 | 2.59 |
| IGLL3P | 2.59 |
| PVRIG2P | 2.59 |
| KRT19P1 | 2.59 |
| ATP5G1P5 | 2.59 |
| PTCHD3P2 | 2.59 |
| FCF1P7 | 2.59 |
| AC010150.1 | 2.59 |
| CTD-2501B8.5 | 2.59 |
| GS1-259H13.7 | 2.59 |
| RPL7P16 | 2.59 |
| AC009238.7 | 2.51 |
| AC009237.17 | 2.51 |
| AC005077.14 | 2.40 |
| IFNWP19 | 2.36 |
| TPSP2 | 2.32 |
| AZGP1P1 | 2.32 |
| HSP90AA6P | 2.32 |
| RPL36AP43 | 2.32 |
| NT5C3AP1 | 2.32 |
| RP11-403I13.10 | 2.32 |
| NACAP3 | 2.32 |
| RP11-205A8.3 | 2.32 |
| RPSAP53 | 2.32 |
| RPL18P10 | 2.32 |
| FER1L4 | 2.32 |
| KB-1269D1.8 | 2.32 |
| PSMD10P2 | 2.27 |
| RP11-757F18.3 | 2.24 |
| PYY | 2.14 |
| RP3-465N24.5 | 2.09 |
| CYP2B7P | 2.06 |
| CDC42P3 | 2.00 |
| RP11-720N19.1 | 2.00 |
| KRT89P | 2.00 |
| ZMYND19P1 | 2.00 |
| PHBP2 | 2.00 |
| CTB-134H23.3 | 2.00 |
| COL6A4P1 | 2.00 |
| KRT17P4 | 2.00 |
| OR10V2P | 2.00 |
| RP11-64K12.1 | 2.00 |
| TSSK5P | 2.00 |
| HMGB3P14 | 2.00 |
| RP11-356M20.1 | 1.99 |
| RP1-34B20.4 | 1.96 |
| TPM3P6 | 1.91 |
| RP11-989E6.13 | 1.88 |
| ACTG1P10 | 1.88 |
| HSPE1P3 | 1.87 |
| LINC00431 | 1.87 |
| RP11-758N13.3 | 1.83 |
| MROH3P | 1.83 |
| SLC35E1P1 | 1.81 |
| RPL12P8 | 1.81 |
| BMPR1APS2 | 1.80 |
| C1DP1 | 1.78 |
| RAD17P1 | 1.74 |
| HIST2H3C | 1.68 |
| HLA-V | 1.66 |
| SLAMF9 | 1.66 |
| CDIPT-AS1 | 1.63 |
| ACTBP13 | 1.59 |
| KRT16P3 | 1.59 |
| KRT18P15 | 1.59 |
| NCAPD2P1 | 1.59 |
| SEPHS1P1 | 1.59 |
| RP11-549B18.3 | 1.59 |
| AQP7P2 | 1.59 |
| SAPCD2P3 | 1.59 |
| ATP5C1P1 | 1.59 |
| LYPLA2P2 | 1.59 |
| TRAPPC13P1 | 1.59 |
| CAP2P1 | 1.59 |
| HNRNPA1P36 | 1.59 |
| UNGP3 | 1.59 |
| AC092641.2 | 1.59 |
| RP11-575G13.2 | 1.59 |
| PCGF7P | 1.59 |
| MTND5P25 | 1.59 |
| NDUFA9P1 | 1.59 |
| RPSAP16 | 1.59 |
| RPL3P9 | 1.59 |
| KRT18P39 | 1.59 |
| RP11-175B9.2 | 1.59 |
| ENPP7P8 | 1.59 |
| TRIM80P | 1.59 |
| RP11-48O20.5 | 1.59 |
| ACTBP9 | 1.59 |
| AP005901.1 | 1.59 |
| FOLH1B | 1.59 |
| MTCYBP21 | 1.59 |
| HIST2H2BC | 1.57 |
| RP11-12A20.6 | 1.56 |
| GUCY1B2 | 1.56 |
| HSPE1P2 | 1.56 |
| RP11-133N21.7 | 1.52 |
| OR7E22P | 1.52 |
| RP11-481A20.10 | 1.49 |
| NUS1P2 | 1.49 |
| ANXA2P1 | 1.48 |
| WTAPP1 | 1.47 |
| HIST1H2AK | 1.45 |
| RP11-295H24.3 | 1.42 |
| AURKAPS1 | 1.42 |
| RP11-395L14.17 | 1.42 |
| ATP5G1P4 | 1.42 |
| CMB9-55F22.1 | 1.41 |
| CHCHD4P3 | 1.37 |
| AADACP1 | 1.36 |
| AK4P1 | 1.34 |
| KRT18P7 | 1.33 |
| NBEAP3 | 1.33 |
| AKR7L | 1.33 |
| ACTG1P15 | 1.33 |
| RP11-424C20.2 | 1.32 |
| CFL1P5 | 1.31 |
| AC068522.4 | 1.31 |
| AC009237.11 | 1.30 |
| NDUFB8P2 | 1.30 |
| RP4-657E11.10 | 1.29 |
| AC005534.6 | 1.27 |
| RNF126P1 | 1.27 |
| RPSAP52 | 1.25 |
| UBA52P6 | 1.25 |
| RP11-27I1.6 | 1.24 |
| ARL4AP2 | 1.24 |
| ARSEP1 | 1.23 |
| CTB-60B18.15 | 1.23 |
| ABCC6P1 | 1.21 |
| FERP1 | 1.21 |
| GTF2IP7 | 1.20 |
| FDX1P1 | 1.20 |
| AC012512.1 | 1.20 |
| EIF4HP2 | 1.19 |
| ZNF887P | 1.18 |
| ZDHHC8P1 | 1.17 |
| RP11-480I12.5 | 1.17 |
| RP13-33H18.1 | 1.16 |
| IGFL1P1 | 1.16 |
| PSMC1P5 | 1.15 |
| RP11-815J4.7 | 1.14 |
| RPL31P52 | 1.13 |
| UBE2CP2 | 1.11 |
| ARMC10P1 | 1.10 |
| SORD2P | 1.09 |
| RP11-381E24.1 | 1.07 |
| IKBKGP1 | 1.06 |
| RP13-401N8.3 | 1.06 |
| RP11-98J23.2 | 1.02 |
| FAHD2P1 | 1.01 |
| AP000344.4 | 1.01 |
| KRT87P | 1.01 |
| ATF4P1 | 1.01 |
| KRT8P41 | 1.01 |
| RP11-54D18.2 | 1.01 |
| TOMM20P4 | 1.01 |
| CTC-471F3.5 | 1.01 |
| ETF1P2 | 1.01 |
| RP11-311D14.1 | 1.01 |
| RP11-747H7.1 | 1.01 |
| RP11-369K16.1 | 1.01 |
| CLCA3P | 1.01 |
| RP11-244J10.1 | 1.01 |
| RP11-635N19.2 | 1.01 |
| KRT18P34 | 1.01 |
| RP13-210D15.8 | 1.01 |
| ZNF833P | 1.01 |
| LRRC37A9P | 1.01 |
| AC097523.1 | 1.01 |
| MGAT4EP | 1.01 |
| CASC4P1 | 1.01 |
| RPS27P25 | 1.01 |
| FBXW11P1 | 1.01 |
| RP11-21G15.1 | 1.01 |
| BEND3P1 | 1.01 |
| KARSP2 | 1.01 |
| TUBG1P | 1.01 |
| ECEL1P1 | 1.01 |
| RP11-44D5.1 | 1.01 |
| CTB-167G5.3 | 1.01 |
| NHP2P1 | 1.01 |
| RPSAP69 | 1.01 |
| RP11-393N4.2 | 1.01 |
| TPTE2P2 | 1.01 |
| BMS1P7 | 1.01 |
| FAM86GP | 1.01 |
| RP11-554D14.2 | 1.01 |
| CTD-2165H16.3 | 1.01 |
| HNRNPA1P27 | 1.01 |
| YWHAEP7 | 1.01 |
| TTC4P1 | 1.01 |
| FAR2P1 | 1.01 |
| DDX3P1 | 1.01 |
| KDELC1P1 | 1.01 |
| CIR1P2 | 1.01 |
| EIF4A2P2 | 1.01 |
| ATF4P2 | 1.01 |
| LILRP2 | 1.01 |
| UBE2SP1 | 1.00 |
| SETP14 | -1.00 |
| RP11-603B24.1 | -1.01 |
| AQP7P1 | -1.01 |
| RP1-278E11.3 | -1.01 |
| SSR4P1 | -1.01 |
| NPM1P25 | -1.01 |
| RP11-848G14.2 | -1.01 |
| RP11-366M4.11 | -1.02 |
| ZNF658B | -1.02 |
| NANOGP7 | -1.02 |
| RPS3AP44 | -1.02 |
| YES1P1 | -1.02 |
| RP11-627K11.1 | -1.02 |
| CEP170P1 | -1.02 |
| RPS15P5 | -1.02 |
| RP11-137N23.1 | -1.03 |
| LYPLA2P1 | -1.03 |
| RP3-425P12.4 | -1.03 |
| POU5F1P3 | -1.03 |
| EIF3KP1 | -1.03 |
| CHCHD2P9 | -1.03 |
| CD8BP | -1.03 |
| EEF1A1P22 | -1.03 |
| SEPT10P1 | -1.03 |
| RP11-488C13.1 | -1.03 |
| RP11-758I14.3 | -1.03 |
| GPS2P1 | -1.03 |
| RP11-64B16.2 | -1.03 |
| RPL13AP3 | -1.03 |
| AL590762.7 | -1.03 |
| RP11-613F7.1 | -1.04 |
| RPS4XP17 | -1.04 |
| RPL7P50 | -1.04 |
| CDC42P4 | -1.04 |
| RP11-530A18.1 | -1.04 |
| RP1-102E24.1 | -1.05 |
| RP11-26H16.1 | -1.05 |
| MTND5P11 | -1.05 |
| EEF1A1P9 | -1.05 |
| ZBTB8OSP2 | -1.05 |
| RAP2CP1 | -1.06 |
| E2F3P1 | -1.06 |
| RP11-486O13.5 | -1.06 |
| CTA-351J1.1 | -1.06 |
| FTH1P16 | -1.06 |
| HSPD1P11 | -1.06 |
| RP11-330M2.4 | -1.07 |
| PFN1P8 | -1.07 |
| EEF1A1P11 | -1.07 |
| RP11-543P15.1 | -1.07 |
| RP11-1267H10.4 | -1.08 |
| CTD-2290P7.1 | -1.08 |
| RP11-401L13.4 | -1.08 |
| RP11-110J1.2 | -1.08 |
| CTD-2262B20.1 | -1.08 |
| FTLP2 | -1.08 |
| RPL7P21 | -1.08 |
| PRKY | -1.08 |
| GAPDHP33 | -1.08 |
| AC078899.1 | -1.08 |
| UNC93B6 | -1.08 |
| AC064836.3 | -1.08 |
| RPL6P27 | -1.09 |
| RPL26P19 | -1.10 |
| DUX4L27 | -1.10 |
| FAM187B2P | -1.10 |
| PGAM1P7 | -1.10 |
| RPL13AP6 | -1.10 |
| RPSAP17 | -1.11 |
| CCDC144CP | -1.11 |
| RAP1AP | -1.12 |
| RP11-353N4.5 | -1.12 |
| BMS1P22 | -1.12 |
| CTC-471J1.10 | -1.12 |
| RP11-673E1.3 | -1.13 |
| RP1-228P16.1 | -1.13 |
| ULK4P2 | -1.13 |
| HMGN2P15 | -1.13 |
| RP4-803A2.2 | -1.14 |
| EEF1A1P4 | -1.14 |
| NOS2P3 | -1.14 |
| RP11-693N9.2 | -1.14 |
| BCORP1 | -1.14 |
| COL6A4P2 | -1.14 |
| RTN3P1 | -1.14 |
| GOLGA6L3 | -1.14 |
| RP11-206P5.2 | -1.14 |
| KNOP1P2 | -1.14 |
| RP11-326L17.1 | -1.14 |
| RP11-779O18.2 | -1.14 |
| RLIMP1 | -1.14 |
| RPL24P8 | -1.14 |
| LINC00264 | -1.14 |
| RPL31P49 | -1.14 |
| KRT223P | -1.14 |
| RP11-298C3.2 | -1.15 |
| USP32P3 | -1.15 |
| ST13P4 | -1.15 |
| RP11-113C12.4 | -1.16 |
| RP11-364L4.1 | -1.16 |
| AC008592.3 | -1.16 |
| ANKRD20A17P | -1.16 |
| RP11-317N8.4 | -1.16 |
| TOMM20P2 | -1.16 |
| RP11-264F23.1 | -1.16 |
| VWFP1 | -1.16 |
| RP11-157G21.2 | -1.17 |
| EEF1A1P19 | -1.17 |
| FCGR2C | -1.17 |
| MTCO3P12 | -1.17 |
| PFN1P4 | -1.18 |
| AC010468.1 | -1.18 |
| RPL23AP49 | -1.18 |
| RPL14P3 | -1.18 |
| RPL7P47 | -1.18 |
| RPS23P6 | -1.18 |
| RP11-29H23.7 | -1.18 |
| RP11-983G14.3 | -1.19 |
| TAS2R18 | -1.19 |
| EEF1GP5 | -1.19 |
| AC010132.11 | -1.19 |
| RP11-262H14.5 | -1.19 |
| RPS8P10 | -1.19 |
| AC104843.3 | -1.20 |
| RPS15AP38 | -1.20 |
| RP11-100N21.1 | -1.20 |
| FAM35DP | -1.20 |
| RP11-25I15.1 | -1.21 |
| RPS4XP2 | -1.21 |
| AC006116.27 | -1.22 |
| RPS20P14 | -1.22 |
| RP11-435F13.2 | -1.22 |
| LGMNP1 | -1.23 |
| BTG1P1 | -1.23 |
| TIMM8AP1 | -1.23 |
| HSPB1P2 | -1.23 |
| NPM1P32 | -1.24 |
| DHX40P1 | -1.24 |
| RPS18P12 | -1.25 |
| RPL12P14 | -1.25 |
| DAP3P2 | -1.25 |
| PFN1P1 | -1.25 |
| RP3-393E18.1 | -1.25 |
| RPL21P7 | -1.26 |
| CDK2AP2P2 | -1.26 |
| DFFBP1 | -1.26 |
| RP11-1094M14.8 | -1.26 |
| RPLP0P2 | -1.27 |
| bP-21264C1.1 | -1.27 |
| RBM8B | -1.27 |
| HMGN1P38 | -1.28 |
| ENPP7P12 | -1.29 |
| RP11-1148O4.1 | -1.29 |
| ANAPC1P1 | -1.29 |
| RP3-340B19.2 | -1.29 |
| RPL21P11 | -1.29 |
| RPL31P11 | -1.29 |
| RP11-395L14.13 | -1.29 |
| SEPT7P9 | -1.29 |
| MXRA5Y | -1.29 |
| RPL7L1P3 | -1.29 |
| TMED10P1 | -1.29 |
| SNX18P12 | -1.29 |
| AC009245.3 | -1.30 |
| RP11-798K23.5 | -1.30 |
| GGTA1P | -1.30 |
| RP11-796G6.1 | -1.30 |
| RP11-203F10.6 | -1.30 |
| RRN3P2 | -1.31 |
| RPL23AP25 | -1.31 |
| AP000936.1 | -1.31 |
| RPS12P21 | -1.31 |
| CTD-2270N23.1 | -1.32 |
| RPL37P2 | -1.32 |
| RPS23P8 | -1.32 |
| RP11-329A14.1 | -1.33 |
| CSPG4P5 | -1.33 |
| RP1-130G2.1 | -1.34 |
| VN1R42P | -1.34 |
| NPM1P9 | -1.34 |
| NME2P1 | -1.35 |
| ALG1L13P | -1.35 |
| ENPP7P2 | -1.35 |
| PLEKHA3P1 | -1.35 |
| AC115617.2 | -1.35 |
| RP11-301M17.1 | -1.35 |
| USP32P1 | -1.36 |
| GPX1P1 | -1.36 |
| CTD-2319I12.10 | -1.36 |
| CYP4F23P | -1.36 |
| RASA4DP | -1.36 |
| DPRXP4 | -1.37 |
| FTH1P11 | -1.37 |
| GGT4P | -1.38 |
| LINC01529 | -1.38 |
| PDLIM1P1 | -1.38 |
| RPL32P1 | -1.39 |
| RP1-241P17.4 | -1.39 |
| CTD-2561J22.2 | -1.39 |
| RP11-76H14.2 | -1.39 |
| RPS26P3 | -1.39 |
| HSPB1P1 | -1.40 |
| GPAA1P1 | -1.41 |
| HIGD1AP11 | -1.41 |
| OR2I1P | -1.41 |
| RP1-40G4P.1 | -1.41 |
| PGBD4P3 | -1.42 |
| CICP18 | -1.42 |
| CYP51A1P1 | -1.42 |
| PYY2 | -1.43 |
| RPL7AP28 | -1.43 |
| RP11-613M5.2 | -1.45 |
| AC006033.22 | -1.45 |
| RP11-707G14.8 | -1.45 |
| TMSB4XP8 | -1.45 |
| CTC-260E6.2 | -1.45 |
| ERVFRD-3 | -1.46 |
| RP13-258O15.1 | -1.46 |
| EIF4A1P4 | -1.46 |
| ACTBP2 | -1.46 |
| RP11-467L13.5 | -1.46 |
| RP11-407P2.1 | -1.47 |
| ESPNP | -1.47 |
| ARPC3P1 | -1.47 |
| RP11-288E14.2 | -1.48 |
| NPM1P21 | -1.48 |
| VN1R85P | -1.48 |
| EEF1A1P29 | -1.48 |
| PPIAP31 | -1.48 |
| RPL36AP26 | -1.49 |
| RP11-151F5.2 | -1.49 |
| RP11-102M11.1 | -1.49 |
| RP11-402J6.3 | -1.49 |
| NAP1L1P1 | -1.51 |
| HLA-DRB6 | -1.51 |
| RP3-423B22.5 | -1.51 |
| RP11-366M4.1 | -1.51 |
| AC079922.2 | -1.52 |
| RP11-109L13.1 | -1.53 |
| FTH1P10 | -1.53 |
| THEMIS3P | -1.54 |
| AGGF1P1 | -1.54 |
| EIF3FP1 | -1.54 |
| EFTUD1P1 | -1.54 |
| KRT16P6 | -1.54 |
| LINC00674 | -1.56 |
| RP11-1134I14.4 | -1.57 |
| bP-2189O9.2 | -1.58 |
| AC013271.3 | -1.58 |
| TRGV7 | -1.58 |
| RP11-587D21.1 | -1.58 |
| ASLP1 | -1.59 |
| NOP56P3 | -1.59 |
| DDX11L2 | -1.60 |
| GYG2P1 | -1.60 |
| RP11-168A11.4 | -1.60 |
| RHOQP3 | -1.61 |
| RPS4XP14 | -1.61 |
| CTD-2224J9.4 | -1.62 |
| YBX1P6 | -1.62 |
| ADAMTS7P1 | -1.62 |
| RP11-360D2.2 | -1.63 |
| BNIP3P17 | -1.65 |
| KRT17P2 | -1.65 |
| RP11-386I23.1 | -1.65 |
| RPL21P119 | -1.66 |
| RP11-848G14.5 | -1.66 |
| RP11-592N21.1 | -1.68 |
| RPL7AP64 | -1.68 |
| XXbac-BPG248L24.12 | -1.68 |
| ACTG1P14 | -1.69 |
| RP11-270C12.3 | -1.70 |
| FTLP3 | -1.70 |
| OR52K3P | -1.71 |
| GGT3P | -1.71 |
| CTD-2248H3.1 | -1.71 |
| RP11-466C23.5 | -1.71 |
| NSFP1 | -1.72 |
| HMGB3P10 | -1.72 |
| RP4-612B18.3 | -1.73 |
| PKD1P5 | -1.74 |
| RP11-182J1.14 | -1.76 |
| GLYATL1P1 | -1.76 |
| EEF1A1P24 | -1.76 |
| RPS3AP25 | -1.76 |
| TMSB4XP6 | -1.77 |
| RP11-974F13.5 | -1.78 |
| RP11-497H16.7 | -1.78 |
| RP11-368J21.3 | -1.79 |
| RP3-476K8.4 | -1.79 |
| ULK4P3 | -1.80 |
| ZNF300P1 | -1.80 |
| FTH1P1 | -1.80 |
| RHOXF1P1 | -1.80 |
| RP11-958N24.2 | -1.81 |
| RP5-903E17.2 | -1.82 |
| HMGB1P1 | -1.83 |
| FTH1P22 | -1.83 |
| ZNF209P | -1.85 |
| AL353898.1 | -1.87 |
| NAPSB | -1.87 |
| SIGLEC22P | -1.88 |
| ADGRE4P | -1.88 |
| RPL21P39 | -1.88 |
| TRAV35 | -1.90 |
| TDH | -1.90 |
| RP11-271K11.5 | -1.92 |
| RP11-16F15.2 | -1.93 |
| RP11-57B24.1 | -1.94 |
| CR848007.2 | -1.94 |
| RP1-159A19.3 | -1.94 |
| DDX11L9 | -1.94 |
| RP11-756A22.7 | -1.98 |
| PTGES3P2 | -2.00 |
| CTD-2287O16.1 | -2.01 |
| COX5BP7 | -2.03 |
| HMGB1P35 | -2.03 |
| GAPDHP32 | -2.03 |
| RPL36P4 | -2.08 |
| ANKRD20A7P | -2.11 |
| RP11-20O24.1 | -2.11 |
| CYP21A1P | -2.11 |
| RP11-484D2.3 | -2.14 |
| RPS26P28 | -2.14 |
| RP11-1036E20.7 | -2.18 |
| IGLV1-41 | -2.20 |
| HNRNPA1P70 | -2.25 |
| AC132008.1 | -2.26 |
| BET1P1 | -2.29 |
| RP11-680E19.2 | -2.29 |
| AC004837.3 | -2.30 |
| RP11-50D9.1 | -2.31 |
| GGTLC5P | -2.31 |
| GAPDHP74 | -2.32 |
| RP11-250H24.2 | -2.34 |
| CTSLP2 | -2.37 |
| SEPT14P2 | -2.37 |
| RP11-331F4.4 | -2.46 |
| RP11-259G18.3 | -2.47 |
| AC005307.3 | -2.50 |
| BNIP3P9 | -2.54 |
| RP11-95M15.2 | -2.55 |
| APOC1P1 | -2.62 |
| RP11-574E24.3 | -2.66 |
| CH507-236L23.2 | -2.76 |
| CH507-338C24.2 | -2.76 |
| IGKV2-29 | -2.77 |
| TMED10P2 | -2.78 |
| RP13-1039J1.2 | -2.79 |
| CTSLP1 | -2.80 |
| RP11-193F5.4 | -2.86 |
| TOP3BP1 | -2.89 |
| RP11-348H3.2 | -2.89 |
| PNLIPRP2 | -3.00 |
| FABP5P3 | -3.12 |
| RP11-241F15.1 | -3.42 |
| NT5CP1 | -3.42 |
| TMED11P | -3.55 |
| CH17-260O16.1 | -3.71 |
| CELP | -3.84 |
| BTF3P10 | -3.90 |
| RP11-562A8.1 | -3.96 |
| RPL23AP21 | -4.67 |

**Table S2**. The expression correlation of AK4P1 with predicted transcription factors (TFs) in PAAD predicted by GEPIA database.

| TF name | R-value | P-value |
| --- | --- | --- |
| YY1 | 0.610 | 0.000 |
| SP1 | 0.510 | 0.000 |
| NF1 | 0.470 | 0.000 |
| VDR | 0.380 | 0.000 |
| IRF2 | 0.330 | 0.000 |
| NR3C1 | 0.300 | 0.000 |
| ELK1 | 0.290 | 0.000 |
| ETS2 | 0.260 | 0.000 |
| RXRA | 0.230 | 0.002 |
| E2F1 | 0.230 | 0.002 |
| IRF1 | 0.230 | 0.002 |
| TBP | 0.210 | 0.006 |
| ETS1 | 0.190 | 0.011 |
| HNF3A | 0.180 | 0.019 |
| LEF1 | 0.160 | 0.028 |
| FOS | 0.150 | 0.047 |
| JUN | 0.099 | 0.190 |
| TP53 | 0.043 | 0.560 |
| HNF1B | 0.036 | 0.640 |
| MAZ | 0.026 | 0.730 |
| PEA3 | 0.023 | 0.760 |
| EGR3 | 0.012 | 0.870 |
| GATA1 | 0.007 | 0.920 |
| GCF | 0.000 | 1.000 |
| PITX2 | -0.001 | 0.990 |
| USF2 | -0.016 | 0.830 |
| NFATC2 | -0.019 | 0.800 |
| NFAT1 | -0.019 | 0.800 |
| HOXD10 | -0.040 | 0.600 |
| XBP1 | -0.047 | 0.530 |
| ESR1 | -0.047 | 0.530 |
| GATA2 | -0.062 | 0.410 |
| NFATC1 | -0.085 | 0.260 |
| FOXP3 | -0.094 | 0.210 |
| POU2F2 | -0.096 | 0.200 |
| HOXD9 | -0.100 | 0.170 |
| HNF1A | -0.110 | 0.140 |
| PAX5 | -0.150 | 0.041 |
| SRY | -0.170 | 0.025 |
| STAT4 | -0.210 | 0.006 |


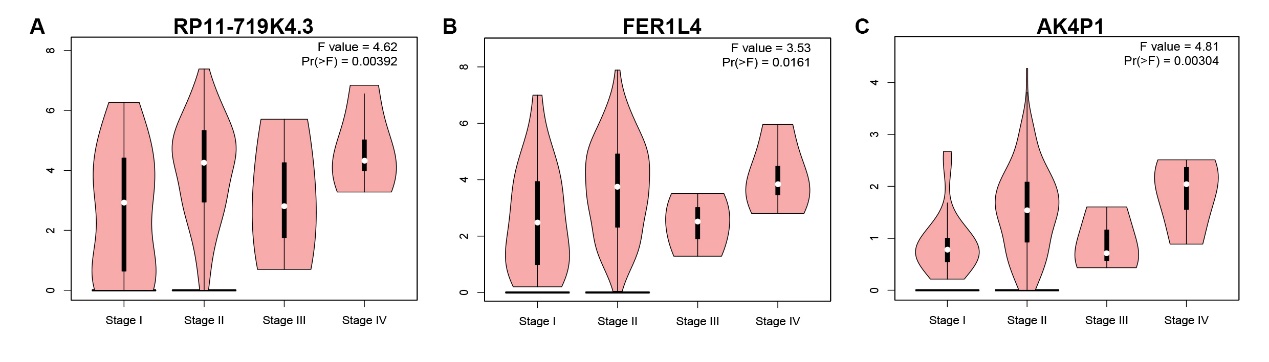


**Figure S1**. The expression differences of three pseudogene-derived RNAs, RP11-719K4.3 (A), FER1L4 (B) and AK4P1 (C) among various major stages in PAAD determined by GEPIA database.


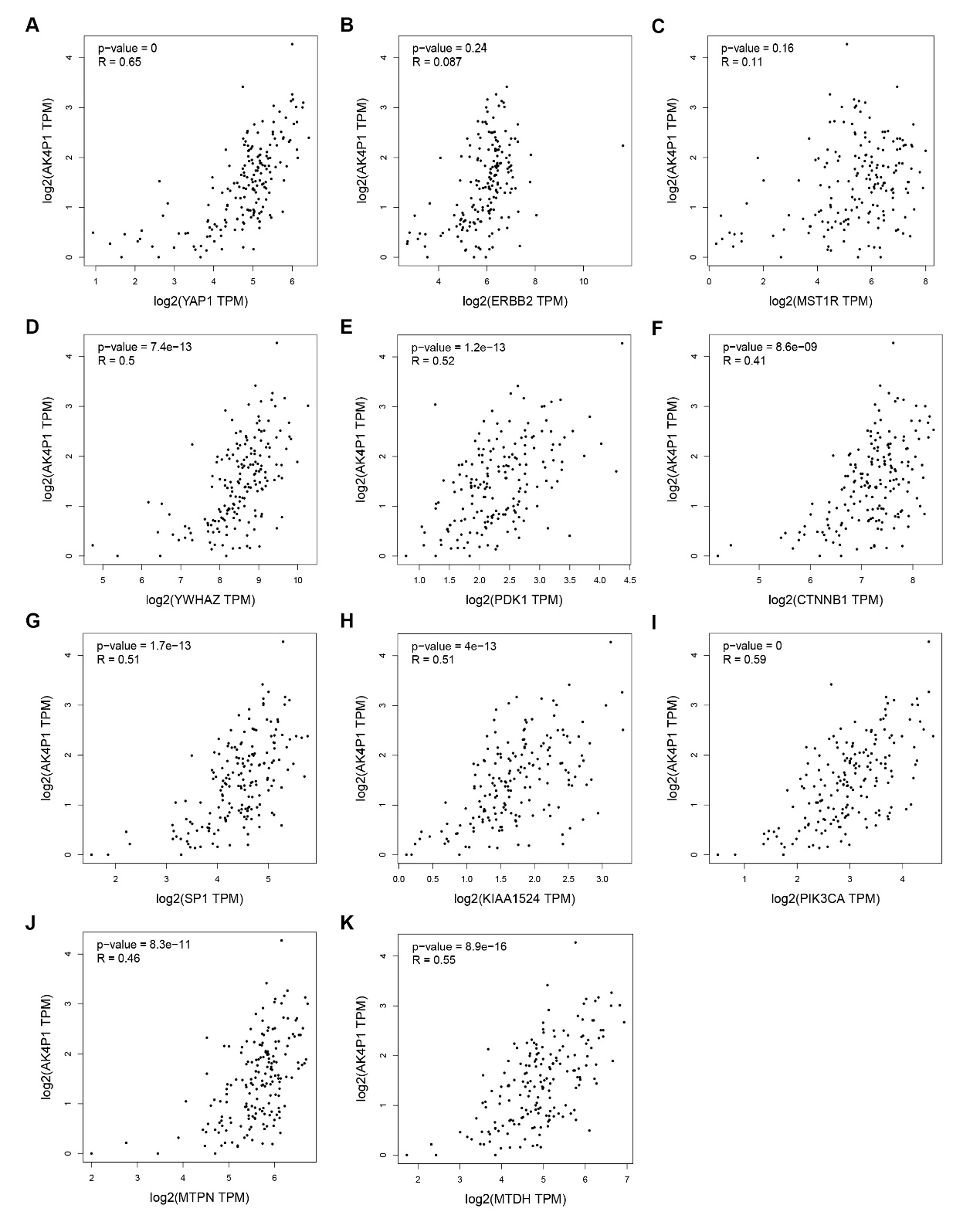


**Figure S2**. The correlation analysis for AK4P1 with 11 target genes of miR-375 in PAAD determined by GEPIA database. The expression correlation of AK4P1 with YAP1 (A), ERBB2 (B), MST1R (C), YWHAZ (D), PDK1 (E), CTNNB1 (F), SP1 (G), CIP2A (H), PIK3CA (I), MTPN (J) or MTDH (K) in PAAD.


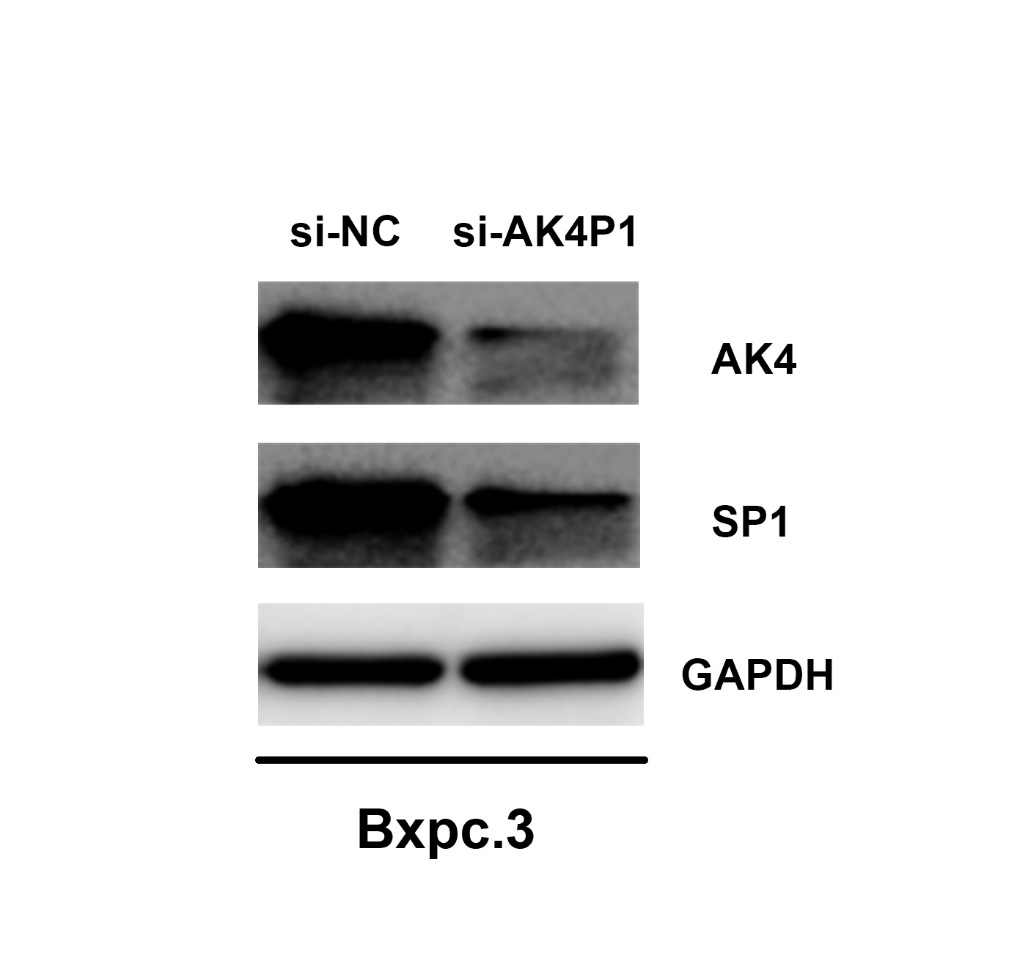


**Figure S3**. The role of AK4P1 in regulating AK4 and SP1 expression in PAAD cell. Knockdown of AK4P1 could significantly downregulate AK4 and SP1 protein levels in Bxpc.3.
